# Identification of genes underlying the enhancement of immunity by a formula of lentinan, pachymaran and tremelia polysaccharides in immunosuppressive mice

**DOI:** 10.1038/s41598-018-28414-w

**Published:** 2018-07-04

**Authors:** Xia Luo, Shaowei Huang, Shuang Luo, Haifeng Liao, Yuanyuan Wang, Xiangliang Deng, Fangli Ma, Chung Wah Ma, Lian Zhou

**Affiliations:** 1 Institute: School of Pharmaceutical Sciences, Guangzhou University of Chinese Medicine, Guangzhou, China; 2 Guangdong Lewwin Pharmaceutical Research Institute Co., Ltd, Guangzhou, China; 3 Infinitus Chinese Herbal Immunity Research Centre, Guangzhou, China

## Abstract

The efficacy of polysaccharides is widespread, especially in immune regulation. However, the genetic basis of the changes in polysaccharides regulating immunity is unclear. To obtain genome-wide insights into transcriptome changes and regulatory networks, we designed a polysaccharide formula, comprising lentinan, pachymaran and tremelia, to increase the availability of their optimized active sites. In this case, we focused on a model of immunosuppression to investigate genes by digital gene expression (DGE) tag profiling in T and B cells. These genes were further validated by qRT-PCR and Western blot experiments. Consequently, polysaccharide formula treatment helped to recover the expression of immune-related genes, including CADM1, CCR2, IGLL1, LIGP1, and FCGR3, FCGR2 in B cells, as well as S100A8, S100A9, ChIL3, MMP8 and IFITM3 in T cells. These results suggest that treatment with polysaccharides improves the immunity of immunosuppressive mice by regulating genes associated with T and B cell functions.

## Introduction

Numerous studies have shown the potency of polysaccharide biological activities, especially in enhancing immunity^1,2^. Polysaccharide treatment not only activates macrophages, lymphocytes, lymphoid factors-activated killer cells, toxic T cells, and dendritic cells (DC) but also promotes the production of cytokines, activates the complement system and accelerates the production of antibodies^3–6^. Among all polysaccharides, those derived from fungus have a wide range of applications^7,8^. For instance, polyporus polysaccharides can inhibit bladder cancer cells^9^, pachymaran can treat tumors in combination with cyclophosphamide(CTX)^10^, and Hericium polysaccharides can alleviate inflammation and ulcers in the digestive tract^11^.

The function of fungal polysaccharides is potent but unique, due to the various junctions between monosaccharides that result in the chemical diversity of these molecules. Different polysaccharides vary in some of their characteristics, including formula weight, branching degree, viscosity, and chain conformation, and these properties strongly affect the biological activities of polysaccharides^12–14^. Additionally, the effect of polysaccharides is closely related to their active components. The main component of polysaccharides is dextran, which is divided into alpha and beta. Alpha glucan is made up of starch, which does not possess immunocompetence in medicine. Beta glucan, which is mainly composed of glucans, such as β-1-3D, β-1-4D, β-1-6D and so on. Beta glucan has proven good effects on tumors, hepatitis and diabetes^15^.

For these reasons, some polysaccharides are combined, and the effect is better than that of a single polysaccharide, and the pharmacological effects of these polysaccharides are synergistic^16^.

In a preliminary study^17^, we found that the formula of multiple polysaccharides was more potent to regulate immunologic activities than each polysaccharide separately. This formula comprised lentinan, pachymaran and tremella, but the mechanisms underlying such effects on immunity remain unclear. To further elucidate the mechanisms underlying the effects of this polysaccharide formula on the immune system, we used immunosuppressive mice and digital gene expression tag profiling to identify genes differentially regulated upon treatment with polysaccharides. We further performed qRT-PCR and Western blotting to confirm the differential expression at both transcript and protein levels. The identified genes enabled us to hypothesize mechanisms underlying polysaccharide treatment and provided a basis for improving immune functions by using the formula of polysaccharides.

## Results and Discussion

### The effects of polysaccharides on immune functions in immunosuppressive mice

The effects of the formula of polysaccharides on immune performance in immunosuppressive mice were investigated, exposuring to cyclophosphamide(CTX) supplemented or no with the polysaccharides.

**Figure 1:**
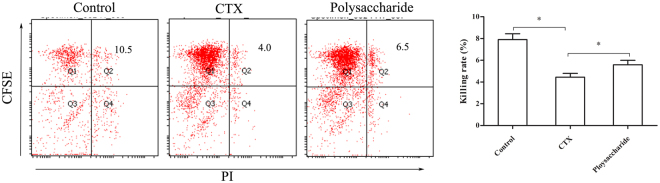
Effects of the polysaccharide formula on killing activity of NK cells in immunosuppressive mice. YAC-1 cells were stained with CFSE as target cells, the dead cells were stained with PI, and CFSE^+^PI^+^ (Q2) were considered killed cells. The figure shows that the compound polysaccharide could improve the killing activity of NK cells. *p < 0.05.

**Figure 2:**
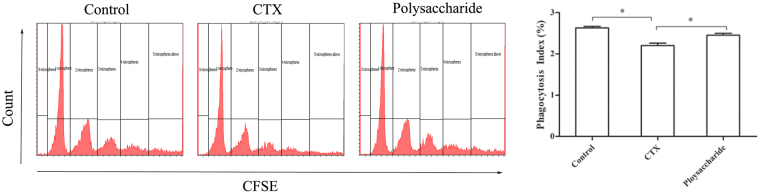
Effects of the polysaccharide formula on macrophage phagocytosis in immunosuppressive mice. The activation of peritoneal macrophage phagocytosis showed with microspheres labeled with CFSE. The first peak on the left shows that the macrophages phagocytosed one microsphere, the second peak shows the phagocytosis of two microspheres, and so on. The figure shows that the compound polysaccharide could improve macrophage phagocytosis. *p < 0.05.

As shown in Fig. 1, the NK cell cytotoxicity in these mice was remarkably reduced by CTX (p < 0.05). In contrast, NK cell cytotoxicity in the polysaccharide-treated group was significantly higher than that in the immunosuppressive group (p < 0.05). The effect of polysaccharides on phagocytosis by peritoneal macrophages is shown in Fig. 2, indicating that the phagocytosis of peritoneal macrophages was markedly inhibited by CTX (p < 0.05). The phagocytosis index of the polysaccharide-treated group was significantly higher than that of the immunosuppressive group (p < 0.05).

**Figure 3:**
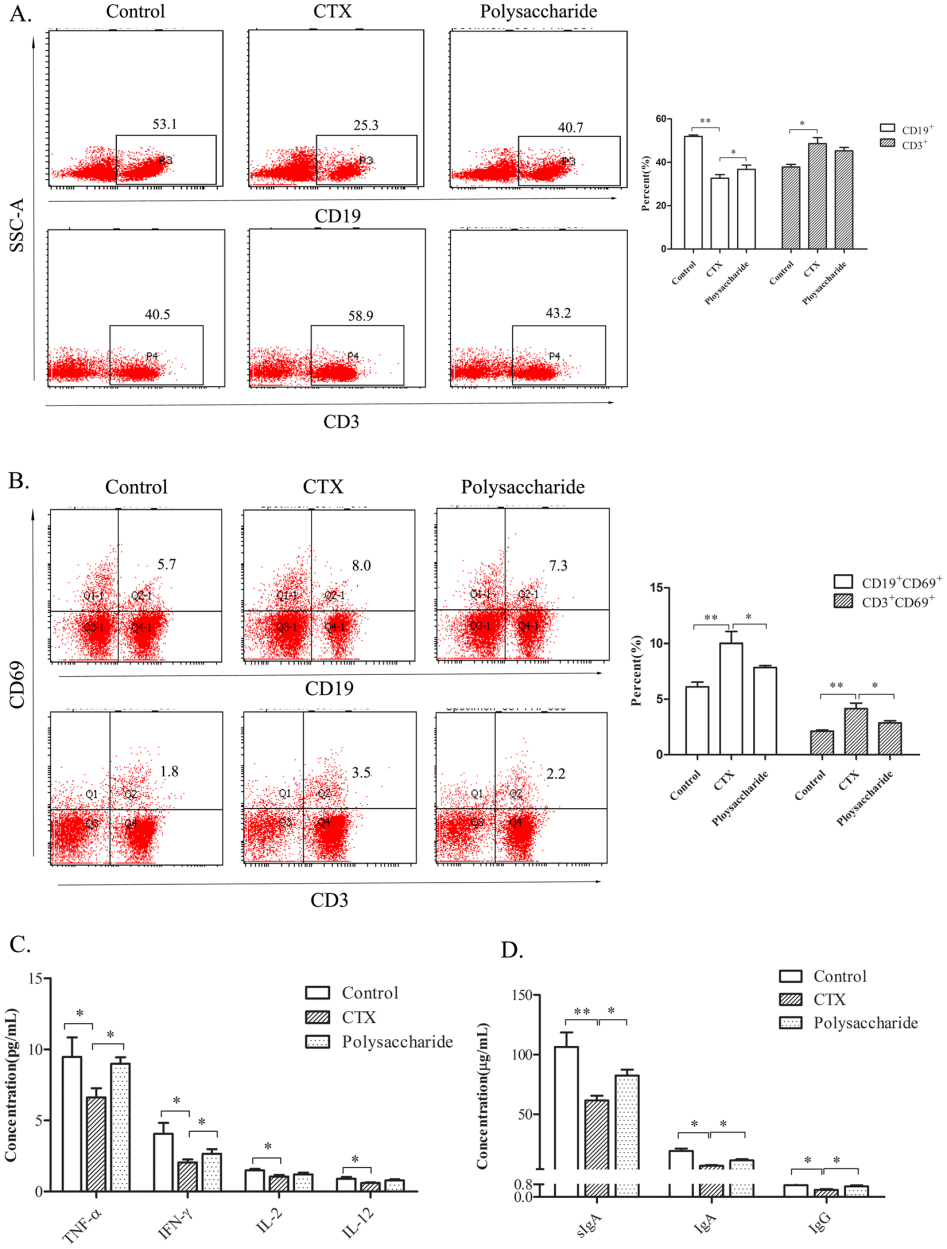
Effect of the polysaccharide formula on lymphocytes. (**A** and **B**) Proportion and activation of T and B cells. Lymphocytes isolated from the spleen were stained with CD3-PEcy7, CD19-PE and CD69-FITC, and analyzed by flow cytometry. CD3^+^ (P4) were considered T cells, CD19^+^ (P3) were considered B cells, CD3^+^CD69^+^ (Q2) were considered activated T cells, and CD19^+^CD69^+^ were considered activated B cells (Q2-1). (**C**) Cytokines in peripheral blood, *P < 0.05. (**D**) Antibody in serum and the small intestine, *p < 0.05.

The effects of polysaccharides on lymphocytes were further investigated. As shown in Fig. 3A,B, the proportion of CD3^+^ (T cell) and CD19^+^ (B cell) spleen lymphocytes was notably unbalanced in mice treated with CTX (p < 0.01), with the proportion of B lymphocytes significantly reduced (p < 0.01) and the proportion of T lymphocytes significantly increased (p < 0.05). As lymphocytes were markedly inhibited by CTX, a compensatory activation of T and B lymphocytes to supplement the lost quantity was observed: CD3^+^CD69^+^ (activated T cells) and CD19^+^CD69^+^ (activated B cells) cells were significantly increased (p < 0.01). However, these changes were significantly reversed in mice fed the formula of polysaccharides (p < 0.05).

The effects of polysaccharides on peripheral blood cytokines are shown in Fig. 3C. The peripheral blood cytokines were significantly reduced after treatment with CTX (p < 0.05). TNF-α and IFN-γ in serum were notably improved in the polysaccharide-treated group when compared with those in the immunosuppressive group (p < 0.05).

The concentration of IgA in the serum and sIgA in the small intestine was significantly reduced by CTX (p < 0.05). In the polysaccharide-treated group, the levels of IgA in the serum and sIgA in the small intestine were notably improved when compared with those in the immunosuppressive group (p < 0.05). (As shown in Fig. 3D).

These experiments demonstrate that treatment with CTX suppresses the immune response, as reflected in the functional decline of macrophages, NK cells, T cells and B cells, which was consistent with the findings of previous studies^18^. Feeding the formula of polysaccharides improved the functions of peritoneal macrophages, NK cells, T cells and B cells in immunosuppressed mice.

### Ingestion of polysaccharides reverses the decrease in immune gene expression

**Figure 4:**
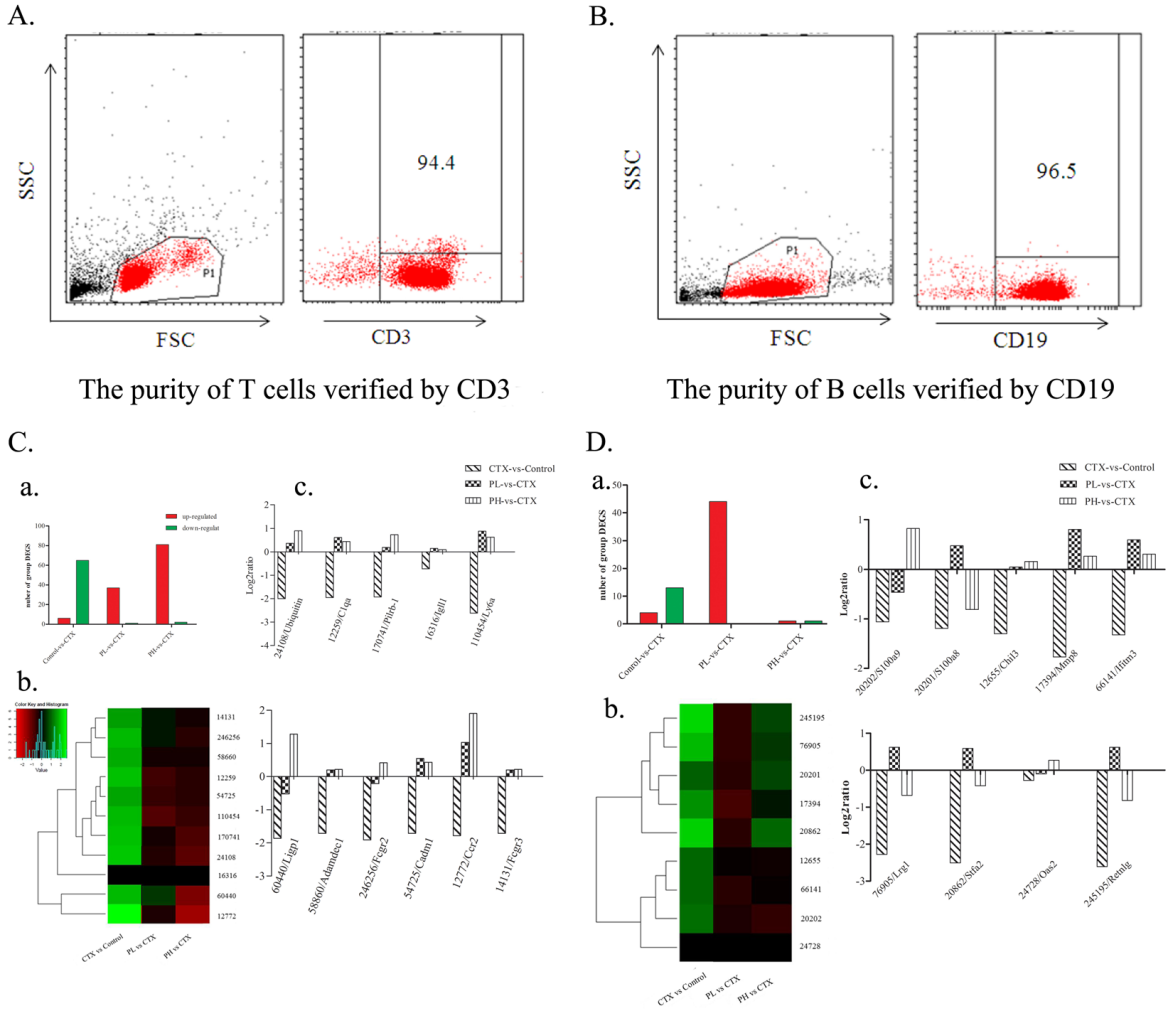
Analysis of the polysaccharide formula on genes in T and B cells in immunosuppressed mice by DGE. (**A**,**B**) Lymphocytes from the spleen isolated by microbeads were stained with CD3-PE, and B220-PE (B cells) to test the purity. The figure shows that the isolated cells are pure. (**C**,a) Quantitative statistics of differentially expressed genes in B cells, the red shows up-regulated genes, and the green shows down-regulated genes. PL is the low dose group of polysaccharides with 200 mg/kg/bw and PH is the high dose of polysaccharide with 400 mg/kg/bw. (b) Hierarchical clustering of differential gene expression in B cells. The clustered map accorded by the log2 of significant difference multiples between the groups. Each row represents a gene and each column represents the comparison between the two groups, the red shows up-regulated genes, and the green shows down-regulated genes, the deeper the color the higher the gene expression. The gene ID is shown on the right, and the enrichment and the function of the displayed genes are shown, with references of the polysaccharide effect on these genes. (c) Statistics on the log2 value of the gene difference multiple in B map. The negative value is down-regulated and the positive value is up-regulated expression, and the 0 indicates no change. The figure shows that the formula of polysaccharide could restore the expression of the following genes in B cells: 24108/*Ubiquitin*, 12259/*C1qa*, 170741/*Pilrb-1*, 16316/*Igll1*, 110454/*Ly6a*, 60440/*Ligp1*, 58860/*Adamdec1*, 246256/*Fcgr2*, 54725/*Cadm1*, 12772/*Ccr2* and 14131/*Fcgr3*. (**D**,a) Quantitative statistics of differentially expressed genes in T cells. (b) Hierarchical clustering of differential gene expression in T cells. The clustered map accorded by the log2 of significant difference multiples between the groups. (c) The figure shows that the compound polysaccharide could restore the expression of the following genes in T cells: 20202/*S100a9*, 20201/*S100a8*, 12655/*Chil3*, 17394/*Mmp8*, 66141/*Ifitm3*, 76905/*Lrg1*, 20862/*Stfa2*, 24728/*Oas2* and 245195/*Retnlg*.

To obtain genome-wide insight into the transcriptome changes in polysaccharides that regulate immunity, we sorted two major immunocytes for DGE experiments. The purities of the sorted T and B cells for the DNA library preparation and expression of sequencing are shown in Fig. 4(A,B). Figure 4C,a shows that treatment with CTX provokes a broad decrease in a number of genes expressed in B (Fig. 4C,a) and T (Fig. 4D,a) lymphocytes and increases the expression of few genes only. Compared with no treatment, treatment with polysaccharides seemed to increase gene expression at both low and high doses in immunosuppressed mice. The top ten genes that were less down-regulated in mice fed polysaccharides upon treatment with CTX (reversion of phenotype) are presented in Fig. 4C,D. Particularly, the expression of genes 24108/*Ubiquitin*, 12259/*C1qa*, 170741/*Pilrb-1*, 16316/*Igll1*, 110454/*Ly6a*, 60440/*Ligp1*, 58860/*Adamdec1*, 246256/*Fcgr2*, 54725/*Cadm1*, 12772/*Ccr2* and 14131/*Fcgr3* was markedly decreased in B lymphocytes (Fig. 4C,b and c), while the compound polysaccharides dose-dependently reversed this down-regulation. In addition, the expression of 20202/*S100a9*, 20201/*S100a8*, 12655/*Chil3*, 17394/*Mmp8*, 66141/*Ifitm3*, 76905/*Lrg1*, 20862/*Stfa2*, 24728/*Oas2* and 245195/*Retnlg* in T lymphocytes was significantly decreased by CTX injection, and the ingestion of polysaccharides partially reversed this effect (see Fig. 4D,b and c).

### qRT-PCR validation of DGE results

**Figure 5:**
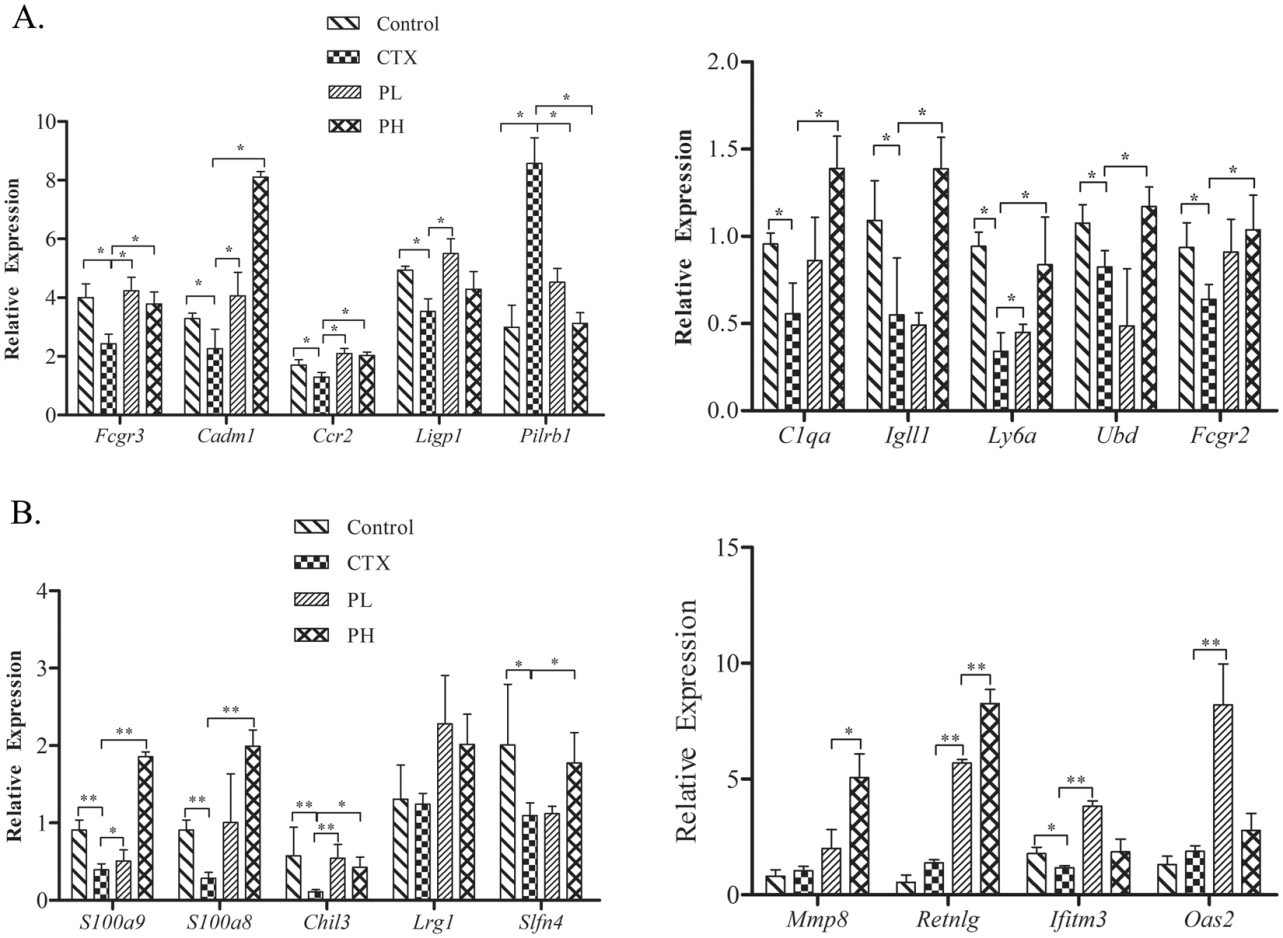
qRT-PCR validation of DGE results of polysaccharides on the genes in T and B cells in immunosuppressed mice. (**A**) The expression of B cell genes. The figure shows that the polysaccharide formula could up-regulate *Fcgr3*, *Cadm1, Ccr2, Ligp1, C1qa, IgllI, Ly6a*, *Ubd* and *Fcgr2* in B cells, consistent with the results of DGE. (**B**) The expression of T cell genes. The figure shows that t the polysaccharide formula could up-regulate *S100a9, S100a8, Chil3, Slfn4*, and *Ifim3* in T cells, consistent with the results of DGE. (*p < 0.05, **p < 0.01).

We next aimed to validate the results of DGE by using qRT-PCR (Fig. 5A,B). The results were consistent with DGE detection, and the verified genes were down-regulated by CTX injection but not in mice treated with polysaccharides. Specifically, treatment with CTX triggered a decrease in the expression of *Fcgr3, Cadm1, Ccr2, Ligp1, C1qa, Igll1, Ly6a, Ubd* and *Fcgr2* in B lymphocytes and *S100a9, S100a8, Chil3, Slfn4*, and *Ifim3* in T lymphocytes. This decrease was significantly reversed in mice exposed to the formula of polysaccharides (p < 0.05).

### Western blot validation of qRT-PCR results

**Figure 6:**
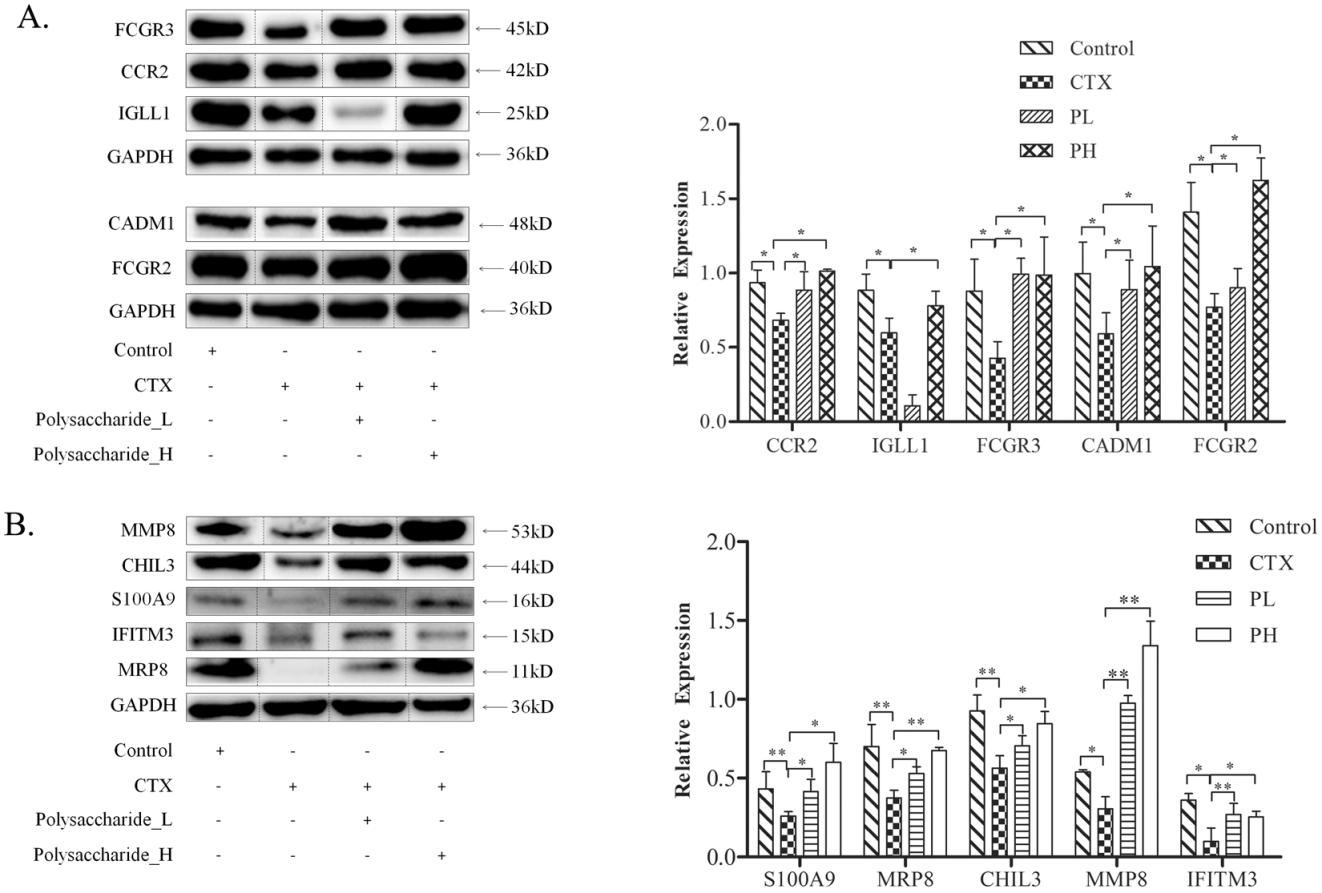
Western blot validation of the DGE results of compound polysaccharides on T and B cell proteins in immunosuppressed mice. (**A**) The expression of B cell proteins. The figure shows that the compound polysaccharide could up-regulate CCR2, IGLL1, FCGR3, CADM1 and FCGR2, consistent with the results of DGE and qRT-PCR. (**B**) The expression of T cell proteins. The figure shows that the polysaccharide formula could up-regulate S100A9, MRP8, CHIL3, MMP8 and IFITM3 in T cells. (*p < 0.05, **p < 0.01).

We finally tested the key proteins expressed by the genes confirmed both by DGE and PCR. Figure 6 shows the changes in protein when treated with polysaccharides after being given CTX in mice. The relative gene expression levels were further quantified (relative amount compared with GAPDH). As shown in Fig. 6A, the relative protein expression of CCR2, IGLL1, FCGR2, CADM1 and FCGR3 was markedly lower in B lymphocytes after treatment with CTX than after the untreated control. Compared with untreated immunosuppressed mice, immunosuppressed mice treated with polysaccharide exhibited a significant increase in the protein levels of CCR2, IGLL1, FCGR2,, CADM1 and FCGR3 (p < 0.05), especially those treated with the high polysaccharide concentration.

Additionally, as shown in Fig. 6B, the relative protein expression of S100A9, MRP8, CHIL3, MMP8 and IFITM3 was significantly lower after treatment with CTX than after treatment with the control (p < 0.05). The relative protein expression of S100A9, MRP8, ChIL3, MMP8 and IFITM3 was significantly higher in polysaccharide-treated mice than in untreated immunosuppressed mice (P < 0.05).

In summary, we screened 20 genes, including 11 genes in B cells and 9 genes in T cells by DGE and verified 8 genes in B cells and 8 genes in T cells by qRT-PCR to show improvements in immune function via polysaccharides. Finally, 10 proteins (5 proteins in B cells and 5 proteins in T cells) expressed by the related genes were reassessed by Western blotting. All of the genes and related proteins validated in the three experiments are shown below, and we speculate that these genes are key points in the regulatory networks that may be involved in polysaccharide improving immunity.

The cell adhesion molecule 1 (CADM1) protein (gene ID 54725) belongs to the immunoglobulin superfamily^19^. CADM1 promotes the cytotoxic effect of NK cells *in vitro*, the secretion of IFN-γ by CD8^+^ cells, as well as NK cell-mediated tumor rejection *in vitro*^19^. CADM1 is also known as tumor suppressor in lung cancer 1 (TSLC1)^20^. Studies have shown the low or missing expression of CADM1 in human tumor tissues and cell lines, including esophageal cancer, cervical cancer, etc.^21–23^. Combined with the effects of polysaccharides on NK cells and cytokine levels in serum, these results suggest that the antitumor activity of the formula of polysaccharides, which involves both NK cells and cytokine production, could be due to its effect on the regulation of CADM1.

Chemokine (C-C motif) receptor 2 (CCR2) (gene ID 12772) is a specific receptor for monocyte chemoattractant protein-1(MCP-1). MCP-1 is an important part of the chemokine network, which serves as chemotactic cue for promoting monocyte/macrophage migration to the lesion^24^. Therefore, CCR2 plays a significant role in anti-infection immunity. The fact that polysaccharides restore the levels of CCR2 and promote the phagocytic activity of abdominal macrophages in immunosuppressive mice, suggests that these compounds could have anti-infection effects, as a consequence of the regulation of CCR2 expression.

Immunoglobulin lambda like polypeptide1 (IGLL1, gene ID 16136) belongs to the immunoglobulin gene superfamily. Mutations in IGLL1 can lead to defects in B cells and gamma globulin hematic disease, which is an autosomal recessive genetic disease^25^. IGLL1 expression is closely related to humoral immunity. Considering that polysaccharides promote the secretion of IgA and sIgA in immunosuppressed mice and increase the expression of IGLL1, these compounds may also improve the humoral immune response by regulating the expression of IGLL1.

The immunoglobulin-receptor CD16/FCGR3 (gene ID 14131) participates in eliminating antigen-antibody complexes in circulation and controls other antibody-dependent reactions. In addition, IgG is encoded by gene 14131^26^. The mutation of this gene can result in virus infection, systemic lupus erythematosus and neonatal autoimmune neutropenia^27,28^. Therefore, combined with the experiment showing that polysaccharides promote the secretion of IgG in immunosuppressive mice it suggests that polysaccharides could alleviate these IgG-associated diseases by regulating CD16/FCGR3.

CD32/FCGR2 (gene ID 246256) is a low-affinity receptor for the immunoglobulin G (IgG) Fc fragment and is primarily expressed on B cells, monocytes, neutrophils, and eosinophils^29^. Many studies have shown that CD32 is correlated with faster HIV progression^30^, suggesting that the formula of polysaccharides could fight HIV infection by regulating CD32/FCGR2.

The S100a8/MRP8 protein (gene ID 20201) belongs to the calcium-binding protein S100 protein superfamily and is involved in the chemotaxis and adhesion of neutrophils. This calcium-binding protein (S100 A8/A9) possesses a wide range of functions, including promoting the circulation of leukocytes and the metabolism of arachidonic acid, as well as the regulation of phagocyte migration or the activation of NADPH oxidases in neutrophils. Therefore, S100A8/A9 plays an important role during the process of inflammation and in anti-infectious immunity^31,32^. Again, the present results suggest that the formula of polysaccharides could bolster anti-infection immunity by modulating the expression of S100 a8/Mrp8.

Similar to S100A8, S100A9 (gene ID 20202) is a member of the calcium-binding protein superfamily and has similar effects^33^, which also suggests that polysaccharides may participate in anti-infection immunity by regulating the expression of S100A9.

Chitinase-like 3 (Chil3, gene ID 12655) catalyzes the conversion of chitin into N-acety1-glucosamine. Chil3 has a wide range of functions, including activating the immune system, preventing metastasis and promoting bacterial clearance^34^. We therefore hypothesized that polysaccharides could increase these functions by regulating Chil3, consistent with the present results showing that the formula of polysaccharides can restore the activation and proportion of lymphocytes in immunosuppressed mice.

Matrix metalloproteinases 8 (MMP8, gene ID 12655) belongs to the metzincin superfamily. Under inflammatory stimulation, MMP8 is released from neutrophils, leading to the degradation of basal membranes and tissue connections to help neutrophils reach inflammatory sites and participate in inflammatory responses, such as lung injury, vascular disease, arthritis, and other inflammatory reactions^35^. MMP8 also plays a role in inhibiting the development of cancers, such as breast cancer, melanoma, tongue cancer, and other cancers^36^. These results suggest that the formula of polysaccharides could exhibit anti-inflammatory and antitumor potentiality by regulating MMP8.

Interferon inducible trans-membrane protein 3 (IFITM3, gene ID 12655) is a double trans-membrane protein belonging to the IFITM family and is primarily stimulated by interferons, participating in cell adhesion, immune, and antivirus activity and inflammatory diseases^37^. We therefore hypothesize that polysaccharides could increase these functions by regulating IFITM3.

Altogether, our results suggest that treatment with CTX induces a decrease in the expression of genes associated with immune function in T and B cells, and polysaccharides stimulate the immune response in immunosuppressed mice by up-regulating the genes primarily involved in complement system, eliminating immune-complex formation, the immunoglobulin cytotoxicity function of NK cells, phagocytosis and so on. We found the genes including 246256/*Fcgr2*, 12259/*C1qa* and 14131/*Fcgr3* were preeminent in these processes and are involved in multiple pathways (see Table 1).

**Table 1:** KEGG pathway enrichment analysis.

Pathway	Pathway ID	All genes with pathway annotation (16857)	P-value	Differentially expressed genes
Cell adhesion molecules	ko04514	223 (1.32%)	0.0101	22329, 54725, 66871, 20970
Chemokine signaling	ko04062	281 (1.67%)	0.0091	12772, 12767, 66871
Primary immunodeficiency	ko05340	50 (0.3%)	9.42213e-05	16136, 12526, 12501
Staphylococcus aureus infection	ko05150	112 (0.66%)	2.150739e-07	12262, 246256, 14289, 12259, 14131
Systemic lupus erythematosus	ko05322	184 (1.09%)	6.912139e-05	12262, 16819, 246256, 12259, 14131, 12260
Natural killer cell mediated cytotoxicity	ko04650	256 (1.52%)	0.0001	15216, 14131, 246256, 22177, 14127, 224840
Fc gamma R-mediated phagocytosis	ko04666	187 (1.11%)	0.0342	246256, 14131, 66871
Phagosome	ko04145	344 (2.04%)	0.0501	246256, 14131, 17533
B cell receptor signaling	ko04662	147 (0.87%)	0.0064	213002, 66141
Transcriptional misregulation in cancer	ko05202	372 (2.21%)	0.0503	98752, 18507, 17394
Amino sugar and nucleotide sugar metabolism	ko00520	82 (0.49%)	0.0161	12655, 12654

The differential genes enriched in KEGG biological pathway were analyzed by bioinformatics analysis tools (p < 0.05 by Fisher’s exact probability test). When p < 0.05, there are changes in biological functions related to certain biological pathways with those differential genes as representatives.

## Conclusion

The present study provided basic genetic information for exploring complex gene expression and the associated regulatory mechanisms of polysaccharides for improving immune functions. These results provide a valuable guide for the diagnosis and treatment of immunosuppressive diseases, especially facilitating the development of new polysaccharides in the future.

## Materials and Methods

### Drugs and reagents

The polysaccharides in the formula were obtained through water extraction and alcohol precipitation according to the routine method, as previously described^38^. Typically, the materials, including *poria cocos (Schw.) wolf*, *tremella fuciformis berk* and *lentinus edodes (Berk.) Sing*, were distilled twice with water, and subsequently filtrated and concentrated, followed by ethanol extraction. The constituents of the polysaccharide formula were detected (see Table 2), and the formula of polysaccharides comprised lentinan, pachynaran and tremelia polysaccharides according to the proportion (lentinan: tremellan: pachymaran = 7:2:1, which was optimized by T cell proliferation experiments^17^).

**Table 2:** Constituents of different polysaccharide in the formula of polysaccharides.

Name of polysaccharide	Polysaccharide(%)	Uronic acid (%)	Protein(%)
Lentinan	74.62	10.44	13.76
Pachynaran	89.96	ND	10.94
Tremelia polysaccharide	69.19	8.12	13.19

The polysaccharide contents were measured by the phenol-sulfuric acid method, and the uronic acid contents were measured by meta-hydroxy-diphenyl, and the protein content was measured with Coomassie Brilliant Blue. ND, not detectable.

### Immunosuppressive mice

BALB/c mice were obtained from the Laboratory Animal Center of Guangzhou University of Chinese Medicine (Guangzhou, China) and acclimated for at least 3 days prior to the experiments. The mice were housed in groups under specific pathogen-free conditions at 24 ± 1 °C, 40–80% humidity, and a 12-h light/12-h dark cycle. All experiments were executed according to the guidelines approved by the Ethics Committee of Guangzhou University of Chinese Medicine. The immunosuppressive mice were induced by CTX (40 mg/kg, i.p.) for 2 days, and indicators to evaluate the model were detected at 5 days after the last injection. Animals in normal control groups were intragastrically administered distilled water for 30 days. Mice in the polysaccharide-treated groups were intragastrically administered the formula of polysaccharides for 30 days (0.2 mL/10 g/bw). For experiments testing immune function, the dose of polysaccharides was 400 mg/kg/bw. For DGE, qRT-PCR and Western blotting, the doses of polysaccharides were 200 and 400 mg/kg/bw.

### Characterization of immune function

The mice were sacrificed by cervical dislocation, and the spleens were immediately removed to prepare lymphocytes (as the effector cells). The cells suspensions were collected and rectified at 5 × 10^6^/mL. The YAC-1 cells (the target cells) were rectified at 2 × 10^6^/mL with PBS, and stained with 2 mol/L CFSE for 10 min. Then, cells were centrifuged and resuspended at 1 × 10^5^/mL in RPMI 1640, and 100 μL suspension was added into a 96-well plate with U-bottom shape (the effector to target ratio is 50:1). All cells were incubated for 2 h at 37 °C, and then collected and centrifuged at 1200 rpm for 10 min. The cells were resuspended in 400 μL PBS and stained with 15 μL PI for 5 min. Stained cells were subjected to flow cytometry (FCM) analysis. The killing activity of NK cells was then calculated with CFSE and PI-double positive cells as the dead target cells. Samples without the YAC-1 cells were controls for natural death.

$${\rm{NK}}\,{\rm{cell}}\,{\rm{activity}}\,( \% )=({\rm{testing}}\,{\rm{group}}\,{\rm{death}}\,\mathrm{rate} \mbox{-} \mathrm{natural}\,{\rm{death}}\,{\rm{rate}})/({\rm{100}}-{\rm{natural}}\,{\rm{death}}\,\mathrm{rate})\times 100 \% $$

The mice received 0.2 mL 2% SRBC (i.p.) on the 26th day to activate macrophages and were sacrificed on the 31st day. Peritoneal macrophages were immediately removed by peritoneal lavage using DMEM. The cells were centrifuged at 1000 rpm for 10 min and resuspended in 1 mL DMEM. Then, 210 μL of fluorescent beads (approximately 1 × 10^9^ beads) were co-incubated with 10 mL of 1% BSA at 37 °C for 30 min, and then subjected to ultrasonic processing for 5 min. The macrophage cell suspension was further co-incubated with 100 μL of a fluorescent bead solution (approximately 1 × 10^7^ beads) for 1.5 h at 37 °C and washed with 2 mL of PBS. The cells were collected in 500 μL of PBS and analyzed with FCM. The analyses were performed by using Dioa software, and the following formulas were used:

$${\rm{Phagocytic}}\,{\rm{index}}={\rm{Number}}\,{\rm{of}}\,{\rm{swallowed}}\,{\rm{fluorescent}}\,\mathrm{beads}/\mathrm{Number}\,{\rm{of}}\,{\rm{counted}}\,{\rm{macrophages}}$$

On the 31th day, the mice were sacrificed and blood was collected from the orbital venous plexus to prepare serum. Additional, the spleens were sterilely removed from the animals to prepare lymphocyte suspensions. The cell suspensions were centrifuged at 1200 rpm for 10 min (supernatant abandoned). The RBC was cracked by disinfected ultrapure water and then hypertonic saline was used to recover to an isotonic state. Lymphocytes were washed twice with 4 mL of PBS and resuspended in 1 mL of RPMI 1640 (containing 10% FBS). The suspensions were filtered with a nylon membrane and rectified at 1 × 10^6^/mL. Then, 400 μL of the suspension for each sample was stained by CD3, CD19 and CD69 (PEcy7/FITC/PE) (eBioscience, San Diego, Calif., USA) and analyzed by FCM. Next, the collected blood was centrifuged at 2000 rpm for 20 min at 4 °C after incubating for 30 min at 4 °C. TGF-β1 in serum was measured with commercial ELISA kits according to the manufacturer’s instructions (Bi Yuntian, Shanghai, China), and TNF-α, IFN-γ, IL-2, IL-6 and IL-12 were detected by a Luminex liquid protein microarray analysis system. The serum was isolated for IgA assy. The small intestines were quickly removed and transferred to a Petri dish filled with 10 mL PBS. The lumen content was flushed and rinsed out with PBS, and the small intestine rinses were collected and centrifuged at 3000 rpm for 5 min. The prepared serum and supernatant were assayed by an ELISA kit for mouse IgA (Xi tang Biotechnology, Shanghai, China).

### Gene expression by digital gene expression (DGE) profiling

The spleen lymphocyte suspensions were prepared as previously described and co-incubated with CD19 or CD90.2 magnetic beads (90 μL per 10^7^ cells) (from Miltenyi Biotec Inc., Germany) at 4 °C, washed with 2 mL of buffer and centrifuged at 300 g for 10 min (supernatants discarded). The cells were resuspended in 500 μL of buffer, and a total of 10^8^ cells were isolated. An MS Separation column was placed into the magnetic field and rinsed with 500 μL of buffer. The cell suspensions were loaded onto the column and washed 3 times with 500 μL of buffer, with the unlabeled cell outflow cupped. Then, the column was removed from the magnetic field and the CD19^+^ (B cells) or CD90.2^+^ (T cells) cells were pushed into the tubes. The purity of the cells was verified by CD3-PE and B220-PE.

The following steps were completed by the BGI Tech Company (Shen Zhen, China). Assorted cells were centrifuged at 300 g for 10 min at 4 °C (supernatant discarded), completely dissolved in 1 mL of TRIzol regent to extract total RNA with a commercial RNA extraction kit. The secondary structure of total RNA was opened by heating, and the mRNA was enriched by magnetic beads with Oligo (dT). Enriched mRNA was fragmented with a moderate amount of interrupting reagent at a high temperature. Using fragmented mRNA as a template, cDNA was synthesized and amplified, and DNA library preparation was accomplished. The quality and quantity (including sample concentration, 28 S/18 S, and RIN) of the prepared library was detected by an Agilent 2100 Bioanalyzer and the ABI StepOnePlus Real-Time PCR System. The eligible library was sequenced by IIluminaHiSeq^TM^ 2000 (performed by Shenzhen Huada Gene Technology).

### Real-Time qPCR

For further validation of the results of DGE, qRT-PCR was performed. The cells were sorted as previously described and total RNA was collected by 1 mL of TRIzol (from Life technologies, California, USA) and stored at −80 °C. RNA was treated with DNase I, and DNase I was subsequently inactivated (incubation at 37 °C for 30 min, 0.5 μL stop solution, then at 65 °C for 10 min). RNA quality was assessed by agarose gel electrophoresis. Then, 1 μg total RNA was placed in a RNase-free PCR tube and DEPC H_2_O was added to 12 μL, followed by mixing and incubation at 85 °C for 5 min. Subsequently, the following reagents were added (on ice): Oligo(dT) 0.5 μL, Random primer 0.5 μL, 10 mM dNTPs 2.0 μL, 5X buffer 4.0 μL, M-MLV 0.5 μL, and RNase inhibitor 0.5 μL (total 20 μL). Finally, reverse transcription was performed at 30 °C, 10 min; 42 °C, 1 h; 85 °C, 10 min. qPCR was performed by using diluted cDNA 2.0 μL, Primer F 0.5 μL, Primer R 0.5 μL, 2X SYBR Green qPCR Super Mix 10 μL (Invitrogen, Carlsbad, CA, USA), and dH_2_O added to 20 μL. The PCR cycles were performed at 50 °C for 2 min, 95 °C for 30 s, and 40 cycles at 95 °C for 5 s and 60 °C for 34 s. The primer sequences are listed in supplementary (Tables 3 and 4).

### Western blotting

To further validate the results of qRT-PCR, Western blotting was performed. The cells were sorted as previously described and lysed in 200 μL of RIPA buffer for 20 min on ice, followed by centrifugation at 12,000 rpm for 10 min at 4 °C. The supernatants were collected. A BCA Protein Assay Kit was used to detect the protein concentration, and the protein samples were mixed with SDS-PAGE loading buffer (5:1), and then boiled for 5 min. The proteins were separated by gel electrophoresis at a constant voltage (80 V) for 50 min, and then at 120 V until the bromophenol blue reached the bottom of the gel. The proteins were then transferred onto a PVDF membrane at 100 V for 60~120 min, and subsequently washed with TBST for 5 min. The membrane was blocked with 5% skimmed milk and then washed with TBST. The blot was incubated with primary antibody [protein marker from Vilnius, Lithuania, antibodies against LRG1, MMP8, Chil3, and MRP8 were purchased from Abcam (Cambridge, Britain), antibodies against Slfn2, S100A9 and RETNLB were purchased from Abnova (USA), and other antibodies were obtained from ABclonal (USA)] at 4 °C overnight or 37 °C for 2 h, and washed 3 times with TBST for 5 min each. Then, the blot was incubated with secondary antibody at 37 °C for 1 h and subsequently washed 3 times with TBST for 5 min each. Finally, the proteins were detected by using an ECL Chemiluminescence Detection Kit HRP (Biyuntian Bio-tech, China).

### Statistical analysis

The experimental data between the two groups were analyzed by using Student’s t-test, and data among several groups were analyzed by one-factor ANOVA in SPSS (version 17.0). A P-value ≤ 0.05 was considered significantly different.

## Electronic supplementary material

Supplementary Information

The primer sequences and abbreviation
