# Supplementary material for: Identification of genes underlying the enhancement of immunity by a formula of lentinan, pachymaran and tremelia polysaccharides in immunosuppressive mice
Source: Sci Rep. 2018 Jul 4;8:10082. doi: 10.1038/s41598-018-28414-w (PMC6031631; doi:10.1038/s41598-018-28414-w)

**Supplemental Figures**

**Title**

Identification of genes underlying the enhancement of immunity by a formula of lentinan, pachymaran and tremelia polysaccharides in immunosuppressive mice

**Authors**

Xia Luo, Shaowei Huang, Shuang Luo, Haifeng Liao, Yuanyuan Wang, Xiangliang Deng, Fangli Ma, Chung Wah MA & Lian Zhou


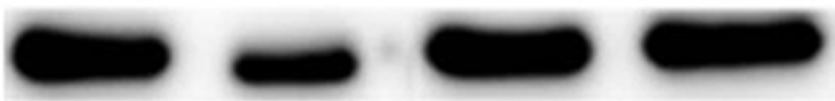

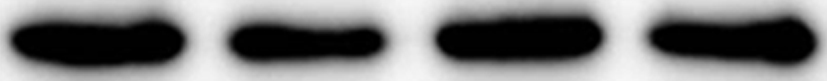

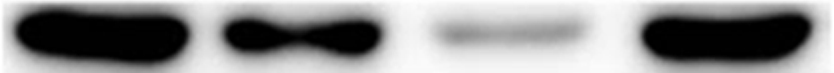

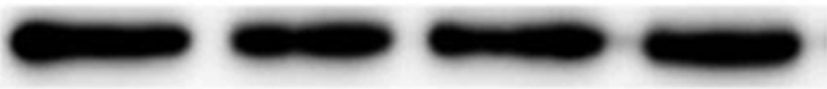


CCR2

IGLL1

GAPDH

42kD

25kD

36kD

FCGR3

45kD

Control

CTX

Polysaccharide_L

Polysaccharide_H

**Supplemental Figure 6A.** The expression of B cell proteins. The proteins of FCGR3, CCR2, IGLL1 and GAPDH were detected by using an ECL Chemiluminescence Detection Kit HRP (Biyuntian Bio-tech, China). The protein of CCR2 is 42kD, near the molecular weight of FCGR3. Accordingly, after CCR2 had been finished detecting, the membrane containing CCR2 and FCGR3 was disposed by Stripping Buffer (CWBIO) and then washed with TBST. After that, the membrane was blocked with 5% skimmed milk, incubated with primary antibody (FCGR3) and following incubated with secondary antibody as the methods mentioned in the manuscript.

Control

CTX

Polysaccharide_L

Polysaccharide_H


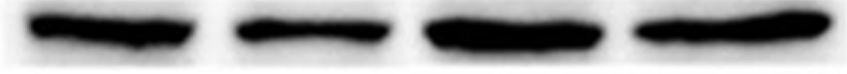

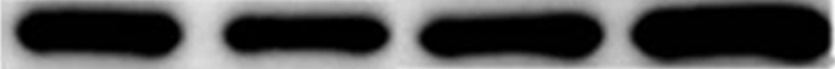

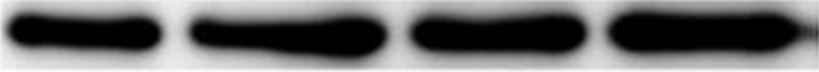


CADM1

FCGR2

GAPDH

48kD

40kD

36kD

**Supplemental Figure 6A.** The expression of B cell proteins. The proteins of CADM1, FCGR2 and GAPDH were detected by using an ECL Chemiluminescence Detection Kit HRP (Biyuntian Bio-tech, China).


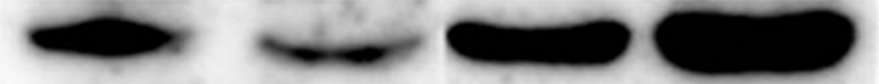

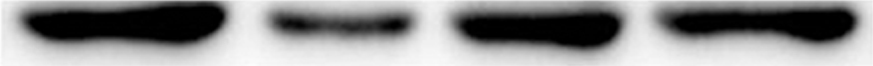

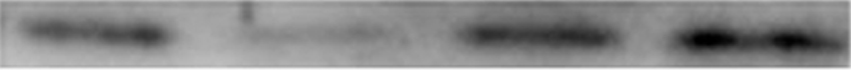

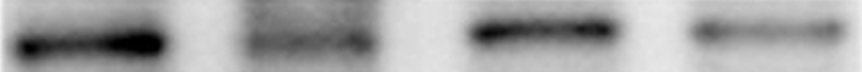

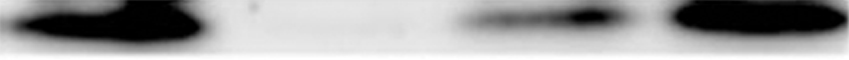

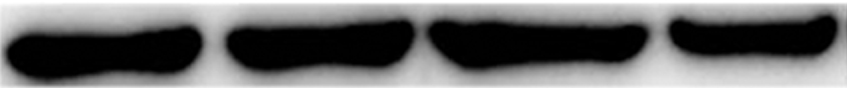


MMP8

CHIL3

S100A9

IFITM3

MRP8

GAPDH

53kD

44kD

16kD

15kD

11kD

36kD

Control

CTX

Polysaccharide_L

Polysaccharide_H

**Supplemental Figure 6B.** The expression of T cell proteins. The protein of MMP8, CHIL3, S100A9 and GAPDH were detected as before. The molecular weight of IFITM3, S100A9 and MRP8 has a little difference, and they were in the same membrane. After S100A9 has been finished detecting, the IFITM3 was disposed as the same as Supplemental Figure 6A, and the protein of MRP8 was detected as before.

**Supplemental Information: Full Blot Images of WB in the Manuscript**

**Title**

Identification of genes underlying the enhancement of immunity by a formula of lentinan, pachymaran and tremelia polysaccharides in immunosuppressive mice

**Authors**

Xia Luo, Shaowei Huang, Shuang Luo, Haifeng Liao, Yuanyuan Wang, Xiangliang Deng, Fangli Ma, Chung Wah MA & Lian Zhou*

**Description**

In the supplemental information, squares with bold lines representing the location of images were used in the main figures.

Fig.6A (2016/04/25)

FCGR3

Control

CTX

Polysaccharide_L

Polysaccharide_H

CCR2


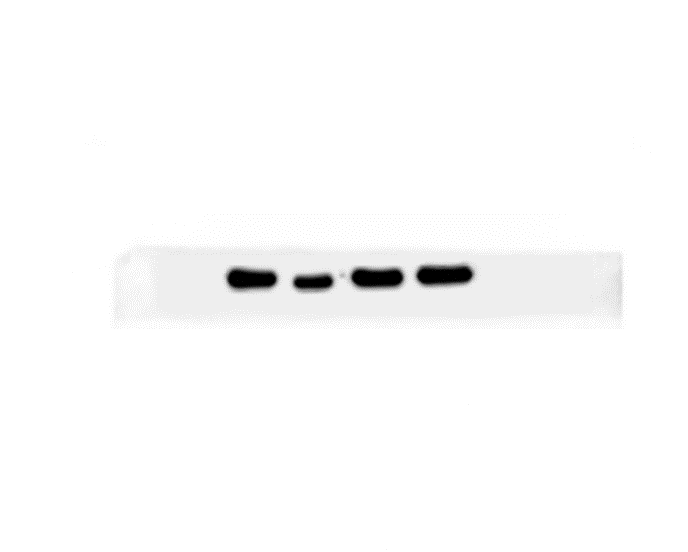

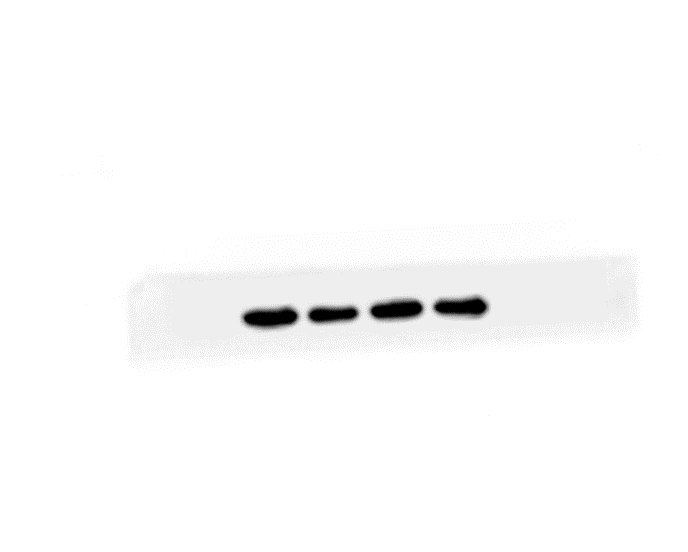

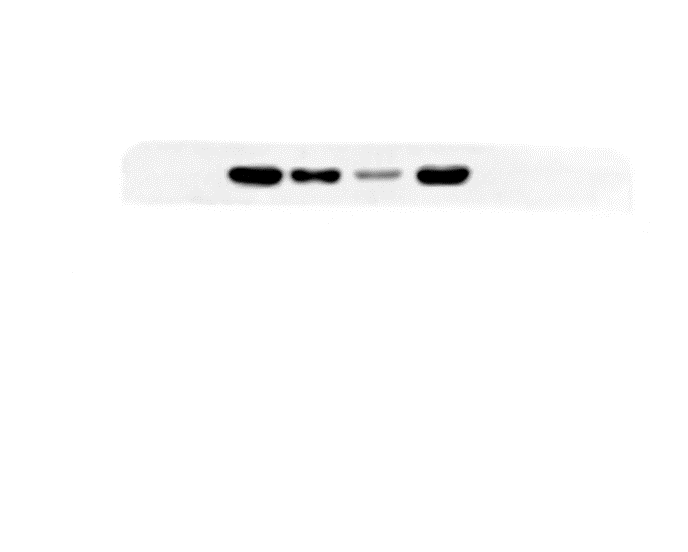


IGLL1


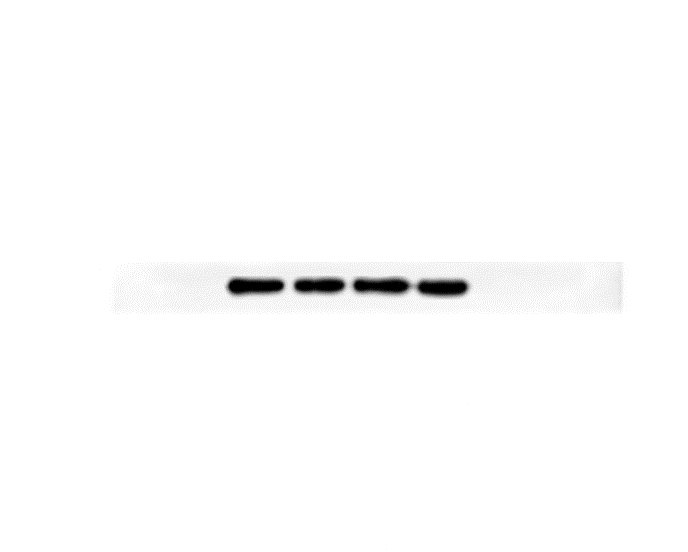


GAPDH

36kD

25kD

42kD

45kD

Fig.6A (2016/04/25)

GAPDH

FCGR2

CADM1


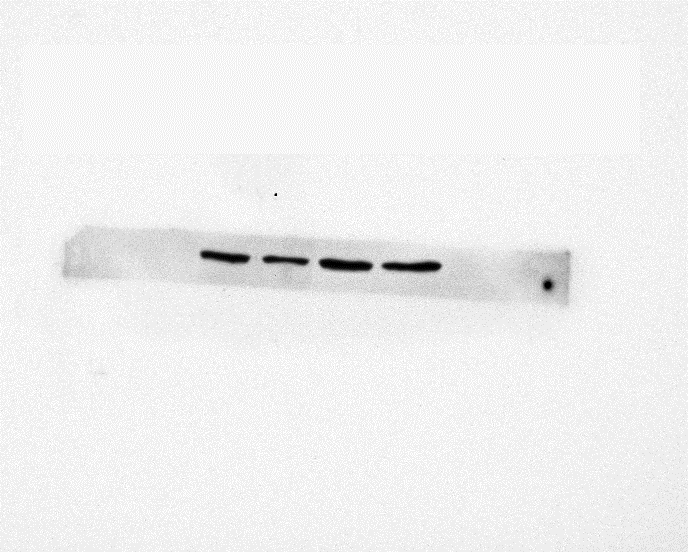

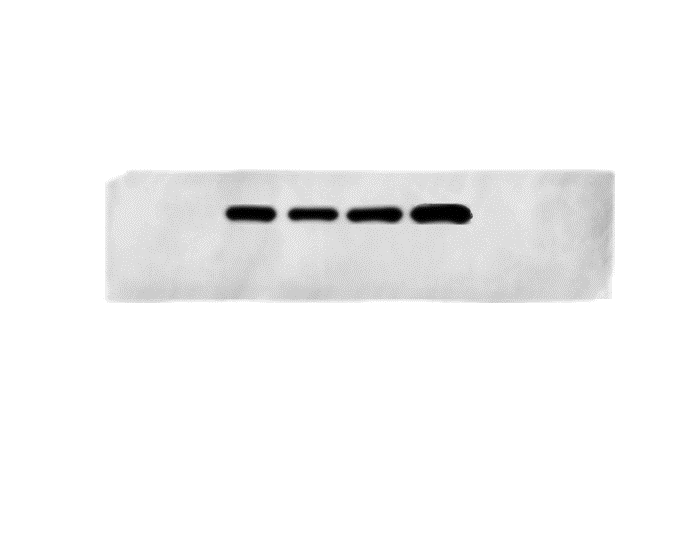

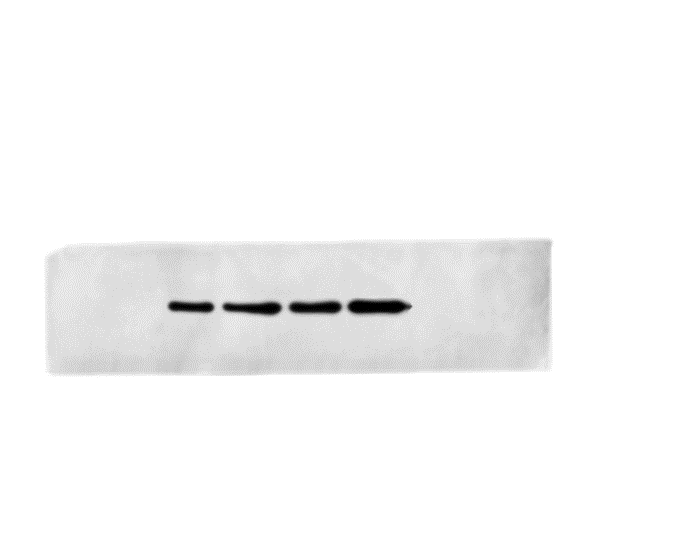


Control

CTX

Polysaccharide_L

Polysaccharide_H

48kD

40kD

36kD

Fig.6B(2016/04/27)


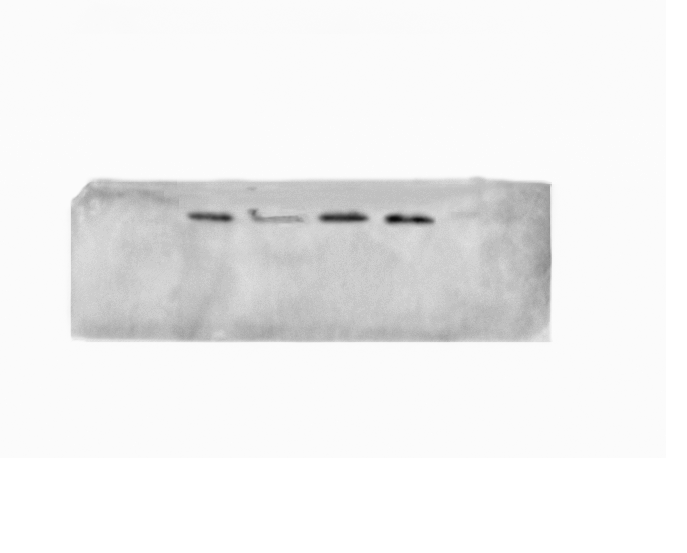

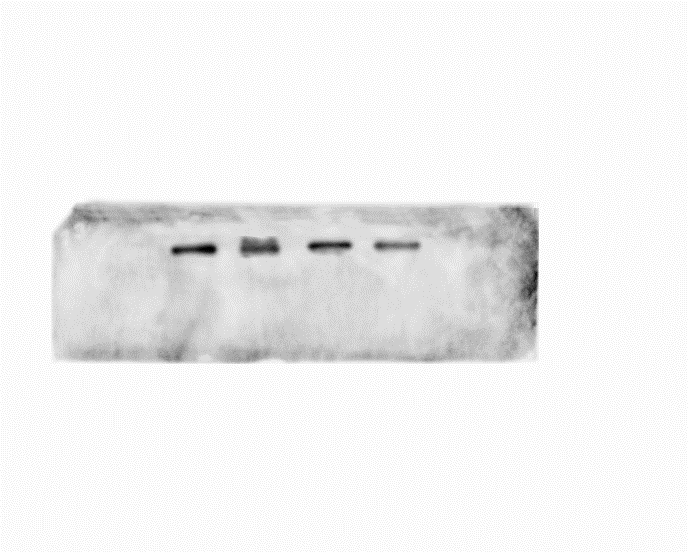

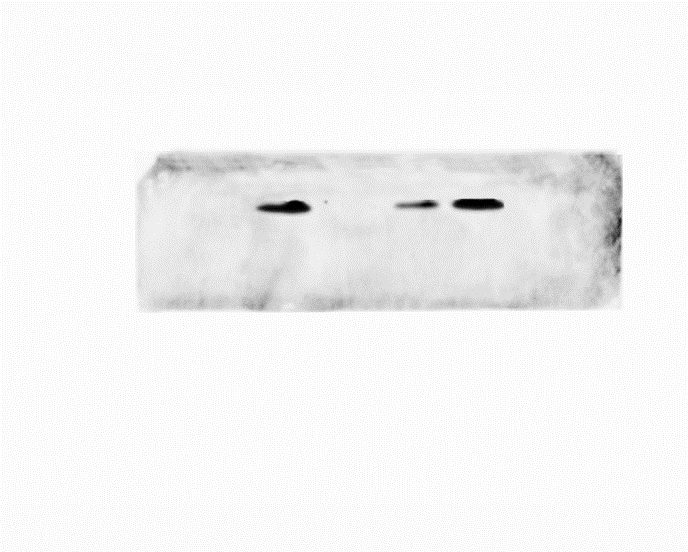


S100A9

IFITM3

MRP8

Control

CTX

Polysaccharide_L

Polysaccharide_H

16kD

15kD

11kD

Fig.6B (2016/04/27)

36kD

44kD

53kD


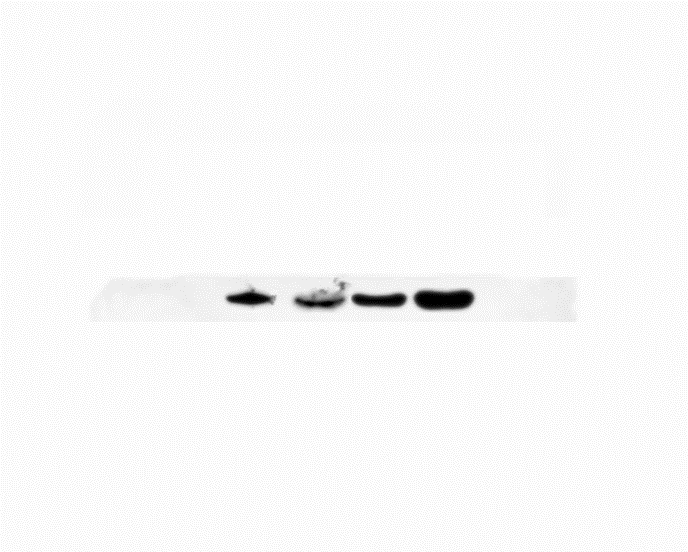

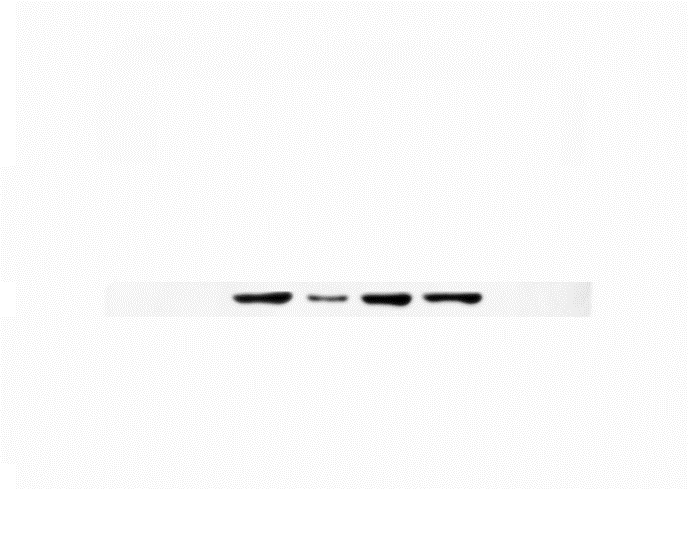


MMP8

CHIL3

GAPDH

Control

CTX

Polysaccharide_L

Polysaccharide_H


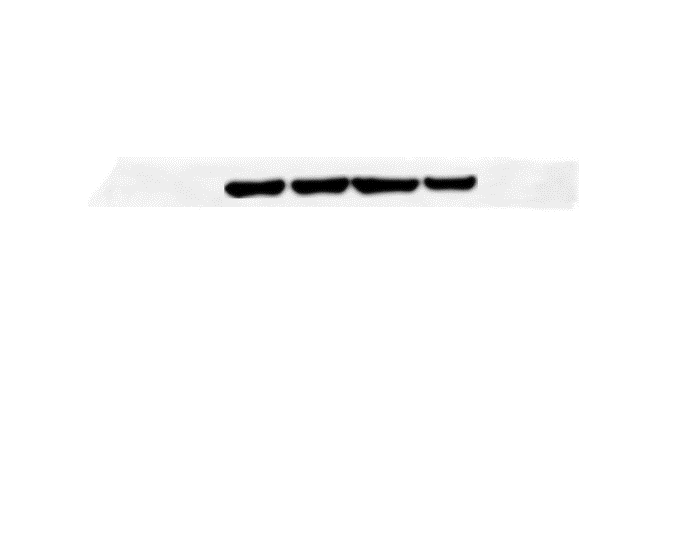

Supplement: Supplementary file 1 — Supplementary Information [file 41598_2018_28414_MOESM1_ESM.docx]
