# Supplementary material for: Identification of genes underlying the enhancement of immunity by a formula of lentinan, pachymaran and tremelia polysaccharides in immunosuppressive mice
Source: Sci Rep. 2018 Jul 4;8:10082. doi: 10.1038/s41598-018-28414-w (PMC6031631; doi:10.1038/s41598-018-28414-w)
Supplement: Supplementary file 2 — The primer sequences and abbreviation [file 41598_2018_28414_MOESM2_ESM.docx]

**Identification of genes underlying the enhancement of immunity by formula of lentinan, pachynaran and tremelia polysaccharide in immunosuppressive mice**

**Xia Luo^1^, Shaowei Huang^1^, Shuang Luo^1^, Haifeng Liao^2^****, Yuanyuan Wang^3^ ,Xiangliang Deng^1,3^, Fangli Ma ^3^, Chung Wah MA^3^ & Lian Zhou^1,4*^**

^1^Institute: School of Pharmaceutical Sciences, Guangzhou University of Chinese Medicine

^2^Guangdong Lewwin Pharmaceutical Research Institute Co., Ltd

^3^Infinitus Chinese Herbal Immunity Research Centre

^4^Corresponding author address: Room B523, Pharmaceutical building, Guangzhou University of Chinese Medicine, No. 232 Outer Ring Road, Panyu District, Guangzhou City, Guang Dong Province, China; e-mail address: zl@gzucm.edu.cn; Zip code: 510006; Tel: +862039358221; Fax: +862039358221

**Table3 The sequence of primers in B cell in Real time PCR**

| **Name of primer** | **Sequence of primer** |
| --- | --- |
| *Fcgr*3-F | 5’-CTGTCCAAGACCCAGCAACT-3’ |
| *Fcgr* 3-R | 5’- GAGCCTGGTGCTTTCTGATT-3’ |
| *Ngp*-F | 5’-ATTGCAGTCGAGAGGATACC-3’ |
| *Ngp*-R | 5’- CAGGATGTTGCCGATGATAT-3’ |
| *Cadm1*-F | 5’-GGAACGGACTGGTTTGTAAA-3’ |
| *Cadm1*-R | 5’- CTAAGGGCTCGGAATAGATG-3’ |
| *Adamdec1*-F： | 5’-GTGATGGGTTGAGAGGGTACT-3’ |
| *Adamdec1*-R | 5’-CGTTTCCTGACAACTTGCT-3’ |
| *Ccr*2-F | 5’-GGAATCCTTGGGAATGAGTAAC-3’ |
| *Ccr*2-R | 5’-CAGGATTAATGCAGCAGTGT-3’ |
| *Ligp*1-F | 5’-TTGCTGCTGACCTAGTGAAT-3’ |
| *Ligp*1-R | 5’-GTCCCTAGCTAATCTCTGCAAA-3’ |
| *Ccr*3-F | 5’-CTGGAAACCCCTTTCTACCG-3’ |
| *Ccr*3-R | 5’-AGGGTCTGTGTGCCAGAATA-3’ |
| *Fcgr*4-F | 5’-CACCAGGATGCCAACTATGT-3’ |
| *Fcgr*4-R | 5’-CCAGCCCATATGGACCTCTA-3’ |
| *Pilrb*1-F | 5’-CTCCCTGCCTTTCATACATG-3’ |
| *Pilrb*1-R | 5’-CCTTCTGTCGACTGCAGATT-3’ |
| *C1qa*-F | 5’-TTCGGCAGAACCCAATGAC-3’ |
| *C1qa*-R | 5’-AGACAAAGGTCCCACTTGGA-3’ |
| *Gadpdh*-F | 5’-GGCCTCCAAGGAGTAAGAAA-3’ |
| *Gadpdh*-R | 5’-GCCCCTCCTGTTATTATGG-3’ |

**Table4 The sequence of primers in T cell in Real time PCR**

| **Name of primer** | **Sequence of primer** |
| --- | --- |
| *S100a*9-F | 5’-AATGGTGGAAGCACAGTTG -3’ |
| *S100a*9*-*R | 5’- CAGCATCATACACTCCTCAAAG -3’ |
| *S100a*8-F | 5’- GCAACCTCATTGATGTCTACCA -3’ |
| *S100a*8*-*R | 5’- GCAAGGAACTCCTCGAAGTT-3’ |
| *Chil*3-F | 5’- CAGCATATGGGCATACCTTT -3’ |
| *Chil*3-R | 5’- CAGACCTCAGTGGCTCCTT -3’ |
| *Oas*2-F | 5’- CGTCCTGGACCTGGTTATAA -3’ |
| *Oas*2-R | 5’- GGTCTGCATTACTGGCACTT-3’ |
| *Slfn*4-F | 5’-TGCCTGGAAAGGTGATATGC-3’ |
| *Slfn*4-R | 5’-AATAGCCCTTTAACTCGGCG -3’ |
| *Ifitm*3-F | 5’-CACTTCTCAAGCCTTCATCAC-3’ |
| *Ifitm*3-R | 5’-GACACCTCTCTGGGCATGTT-3’ |
| *Mmp*8-F | 5’-TGGCTGCTCATGAATTTGGA-3’ |
| *Mmp*8-R | 5’-TGGGCCCAGTAGGTTGGAT-3’ |
| *Lrg*1-F | 5’- GAAGCCTCCAGGATCTCAAG -3’ |
| *Lrg1*1-R | 5’-GAGAATTCCACCGACAGATG-3’ |
| *Stfa*2-F | 5’- GACCTGCCACATCAGAAA-3’ |
| *Stfa*2-R | 5’- TTGGACGACTTGAACTTTAT-3’ |
| *Retnlg*-F | 5’- AGGAACTTCTTGCCAATCG-3’ |
| *Retnlg*-R | 5’- GCCTGAAGCCGTGATACT-3’ |
| *Gadpdh*-F | 5’-GGCCTCCAAGGAGTAAGAAA-3’ |
| *Gadpdh*-R | 5’-GCCCCTCCTGTTATTATGG-3’ |

**Table5 Abbreviation**

| **Abbreviation** | **Amplification** |
| --- | --- |
| BSA | bovine serum albumin |
| CADM1 | cell adhesion molecule1 |
| Chil3 | chitinase-like 3 |
| DEPC | diethypyrocarbonate |
| DGE | Digital Gene Expression |
| ELISA | enzyme linked immunosorbent assay |
| FBS | fetal bovine serum |
| FCGR3 | Fc-gamma RIII |
| FCM | flow cytometer |
| ID | identification |
| IFITM3 | interferon-induced transmembrane protein3 |
| IFN-γ | Interferon-γ |
| IgG | Immunoglobulin-G |
| IGLL1 | Immunoglobulin lambda like polypeptide1 |
| IL-2 | interleukin-2 |
| LRG1 | leucine-rich-alpha-2-glycoprotein1 |
| MRP8 | myeloid-related protein-8 |
| NK | natural killer |
| PVDF | polyvinylidene fluoride |
| RT-qPCR | Real-time quantitative polymerase chain reaction |
| SRBC | sheep red blood cells |
| TGF-β1 | transforming growth factor-β1 |
| TNF | tumor necrosis factor |
